# Estimating the Duration of Public Concern After the Fukushima Dai-ichi Nuclear Power Station Accident From the Occurrence of Radiation Exposure-Related Terms on Twitter: A Retrospective Data Analysis

**DOI:** 10.2196/publichealth.5384

**Published:** 2016-11-25

**Authors:** Naoki Nishimoto, Mizuki Ota, Ayako Yagahara, Katsuhiko Ogasawara

**Affiliations:** ^1^ Kagawa University Hospital Clinical Research Support Center Kita-gun Japan; ^2^ Hokkaido University Graduate School of Health Sciences Sapporo Japan; ^3^ Faculty of Health Sciences Department of Radiological Technology Hokkaido University of Science Sapporo Japan; ^4^ Hokkaido University Faculty of Health Science School Sapporo Japan

**Keywords:** Twitter, social media, public concern, nuclear power plants, survival analysis, Kaplan-Meier estimate, infodemiology, radiation

## Abstract

**Background:**

After the Fukushima Dai-ichi Nuclear Power Station accident in Japan on March 11, 2011, a large number of comments, both positive and negative, were posted on social media.

**Objective:**

The objective of this study was to clarify the characteristics of the trend in the number of tweets posted on Twitter, and to estimate how long public concern regarding the accident continued. We surveyed the attenuation period of the first term occurrence related to radiation exposure as a surrogate endpoint for the duration of concern.

**Methods:**

We retrieved 18,891,284 tweets from Twitter data between March 11, 2011 and March 10, 2012, containing 143 variables in Japanese. We selected radiation, radioactive, Sievert (Sv), Becquerel (Bq), and gray (Gy) as keywords to estimate the attenuation period of public concern regarding radiation exposure. These data, formatted as comma-separated values, were transferred into a Statistical Analysis System (SAS) dataset for analysis, and survival analysis methodology was followed using the SAS LIFETEST procedure. This study was approved by the institutional review board of Hokkaido University and informed consent was waived.

**Results:**

A Kaplan-Meier curve was used to show the rate of Twitter users posting a message after the accident that included one or more of the keywords. The term Sv occurred in tweets up to one year after the first tweet. Among the Twitter users studied, 75.32% (880,108/1,168,542) tweeted the word radioactive and 9.20% (107,522/1,168,542) tweeted the term Sv. The first reduction was observed within the first 7 days after March 11, 2011. The means and standard errors (SEs) of the duration from the first tweet on March 11, 2011 were 31.9 days (SE 0.096) for radioactive and 300.6 days (SE 0.181) for Sv. These keywords were still being used at the end of the study period. The mean attenuation period for radioactive was one month, and approximately one year for radiation and radiation units. The difference in mean duration between the keywords was attributed to the effect of mass media. Regularly posted messages, such as daily radiation dose reports, were relatively easy to detect from their time and formatted contents. The survival estimation indicated that public concern about the nuclear power plant accident remained after one year.

**Conclusions:**

Although the simple plot of the number of tweets did not show clear results, we estimated the mean attenuation period as approximately one month for the keyword radioactive, and found that the keywords were still being used in posts at the end of the study period. Further research is required to quantify the effect of other phrases in social media data. The results of this exploratory study should advance progress in influencing and quantifying the communication of risk.

## Introduction

### Spreading Concern About Radiation Exposure: Social Media and the Fukushima Dai-ichi Nuclear Power Station Accident in Japan

When the Fukushima nuclear power station accident occurred in Japan on March 11, 2011, concerns about radiation exposure arose. Continuous anxiety was assumed to affect people’s physical and mental health, and the duration of fear or anxiety regarding the accident has remained unknown. An accurate estimation of the duration of concern would lead to recommendations related to public health action. To date, the duration of public concern has been difficult to measure because the data can be multi-scaled according to various topics and time scales. Many comments regarding the Fukushima accident have been posted on social media, such as Facebook and Twitter. It has become apparent that social media platforms encourage people to share their concerns regarding radiation exposure in their daily lives; a person’s concern is highly correlated with the amount and length of a communication [1]. Thus, our primary interest was to quantify the amount of communication related to the specific topic of radiation exposure. Quantifying public anxiety plays an important role in public interventions [2].

Slovic surveyed the risk perception of lay people and experts concerning 30 activities, including nuclear power, and conducted factor analysis by plotting 81 hazards on a chart with the axes *dread risk* and *unknown risk* [3]. The concept *diagnostic X-rays* showed a low dread risk value, whereas *nuclear reactor accidents*, *nuclear weapons fallout*, and *radioactive waste* had high values for dread risk and medium values for unknown risk [3]. Slovic mentioned the Three Mile Island nuclear accident and concluded that the perception of risk was not related to the estimated social cost [3].

Mehta and Simpson-Housley reported a linear formula composed of factors such as trait-anxiety scores, gender, and whether people had children [4]. This research showed that expectation of a future nuclear power plant disaster was positively associated with high trait-anxiety scores, the female gender, and having children living in the household [4]. Until recently, it has not been possible to establish the effect of social media in generating public anxiety about nuclear power plant accidents and radiation exposure from radioactive waste. However, we have not been able to find any previous studies regarding the traits of terminology to capture written concerns about nuclear power plant accidents.

Exploring Twitter data, we aimed to generate a hypothesis regarding Twitter users’ interest related to the Fukushima Dai-ichi Nuclear Power Station accident. Focusing on Twitter users, we surveyed previous research on how users express their concerns regarding radiation exposure or a power station accident, and found only a small number of articles in the psychological literature [5]. With the emergence of social media, Twitter users have played a central role in health and the health care process, not only as recipients of health services, but also as initiators of positive personal health action. Users also bring the patient community together (eg, PatientsLikeMe.com, whose members manage home-based care for themselves and others) and act as citizens engaged in collaborative practices (ie, promoting proper sanitization and clean air) to ensure the health of their communities [6,7].

### Social Media in Health Care Research

Previous studies have explored evidence regarding the effects of social media on the perceptions of Twitter users. Approximately 90% of the Dutch population aged 12 years and older use the Internet, and 70% of these individuals are active on social media, particularly Facebook and Twitter (ie, the Web 2.0) [8]. According to the Japanese Ministry of Internal Affairs and Communications’ *Communication Usage Trend Survey 2015*, Internet usage in Japan has shown an increasing trend, with approximately 82.2% of the population using the Internet and 62.6% using smartphones [9].

Twitter was introduced as an education support tool in the nursing domain for the first time in 2006 [10-13]. The neologism *infoveillance* (information + surveillance) was introduced by Eysenbach [14,15]. Novel methods for infoveillance are becoming available, such as mining, aggregating, and analyzing online textual data in real-time. Studies have shown that pandemic predictions or estimates of influenza-like illnesses derived from Twitter accurately track the reported levels of diseases [16-18]. Using Twitter data in conjunction with epidemiological data, Chew and Eysenbach reported on pandemic prediction of the H1N1 influenza outbreak, using double plot analyses to demonstrate that sharp increases in the absolute volume of H1N1-related tweets coincided with major H1N1 news events [18]. This finding demonstrated the usefulness of infodemiology techniques for pandemic prediction. In their 2014 study, Zhao et al reported a method for identifying influential users from Twitter data [19]. Several social media platforms are currently available, such as Twitter, Facebook, and LinkedIn. Due to its popularity, we focused on Twitter in this study.

### The Problem of Social Media-Based Big Data in Health Care

The concept of *big data* was introduced to the public by Douglas Laney, who discussed the problem of big data with respect to data volume, velocity, and variety [20]. At the time Laney wrote about this issue, big data had for the first time become widespread in e-commerce; it subsequently infiltrated health care. The current definition of big data includes a further term: veracity [21]. Big data was regarded as a combination of large-scale, structured, and unstructured data, and the digital record of social infrastructure or social media was also regarded as part of this. The United States Federal Government has announced the challenges of big data and its mission for a big data program, which includes, “Informatics for Integrating Biology and the Bedside” developed by the National Library of Medicine, with the aim of creating more than 50 tools and approaches to facilitate the integration and exchange of informational byproducts of health care and biomedical research.

These tools were developed through open source sharing. Two major problems remain with this project. First, big data involves a large-scale volume of data, and powerful computing is required to handle this, yet professional medical staff, for example, may require real-time access to the data. Laney proposed the selection of data through sampling [20]. The American Statistical Association (ASA) published their policy entitled, “Discovery with data: leveraging statistics with computer science to transform science and society” to address these problems [21]. To handle large-volume data, the ASA reviewed techniques and started with data visualization. The statistics community has a long history of developing data visualization techniques, not just histograms and scatterplots, but also techniques such as trellis plots and dynamic graphs. The ASA has also introduced modern visualization techniques such as treemaps and other techniques for visualizing network data; these approaches are going to be heavily in demand, and new ways of visualizing complex data with specific properties will need to be developed [22]. Causal inference is also covered in the policy of the ASA, and summarizing data using graphs plays an important role in estimating causal inference. Techniques used in data mining do not appear to be synthesized with conventional statistical methodology; the ASA has proposed combining data mining techniques with visualization, and have claimed that this approach has the potential to exceed the power of either field alone.

Second, it is difficult to identify the individual who posted a comment on social media, and existing statistical methodologies can be difficult to use, as it may not be clear whether the observation was independent or not. The problem of defining a *study population* remains in social media research, as some people post comments using a user identification (ID) with multiple users, or a user ID from an automated tweeting program. Twitter has a growing user population and is open in nature, which has made it an ideal target for exploitation by automated tweeting programs, known as *bots*. Like bots in other Web applications such as Internet chatrooms, blogs, and online games, bots have become common on the Twitter platform [23-25].

### Motivation

We have focused on public concern related to the risk of a nuclear power plant accident and concern about the negative effects of radiation on the human body. Public concern is affected by the media (eg, TV, radio, or Internet), making this study unique from an informatics perspective. Over the course of this study we observed a widespread concern that has not been quantified. Some phrases related to radiation exposure (ie, *air dose rate* or *no radiation particle spread*) that were mentioned in microblogs may have been used to infer the status and safety of specific conditions in geographically distinct regions. However, it is not well established when these words arise or how long public concern lasts among Twitter users.

### Purpose

The duration of public concern has thus far been difficult to measure, because the data can be multi-scaled according to various topics and time scales. Exploring Twitter data required making assumptions regarding Twitter users’ interest in the Fukushima Dai-ichi Nuclear Power Station accident.

The purpose of this study was to clarify the characteristics of the trend in the number of tweets posted on Twitter, and to estimate how long public concern about the accident lasted. We surveyed the attenuation period of radiation exposure-related terms for the first time as a surrogate endpoint for the duration of concern.

## Methods

### Twitter Data and Tools

We retrieved Japanese Twitter data from March 11, 2011 to March 10, 2012 as social networking service data from Twitter, Incorporated [26]. Twitter was created in March 2006, and the service rapidly gained worldwide popularity; by 2012, more than 100 million users were posting 340 million tweets per day [27]. Each tweet contains a Tweet ID, a textual part (restricted to 140 characters), and the tweet’s date and time. We obtained 18,891,284 tweets and 143 variables, including date and time of tweet, tweet text, user ID, and user names. The file size totaled 17.2 gigabytes. The data was in comma-separated values (CSV) format.

We also used the programming language Java (jre1.8.0_25) with Eclipse Luna 4.4.0, an integrated development environment, for data handling. The statistical packages SAS 9.4 and JMP 11 pro (both SAS Institute Inc., Cary, NC) were used for estimating the survival curve. We used the HP Z420 workstation (Hewlett-Packard Company) with 3.7 gigahertz central processing unit and 64 gigabyte memory.

**Figure 1:**
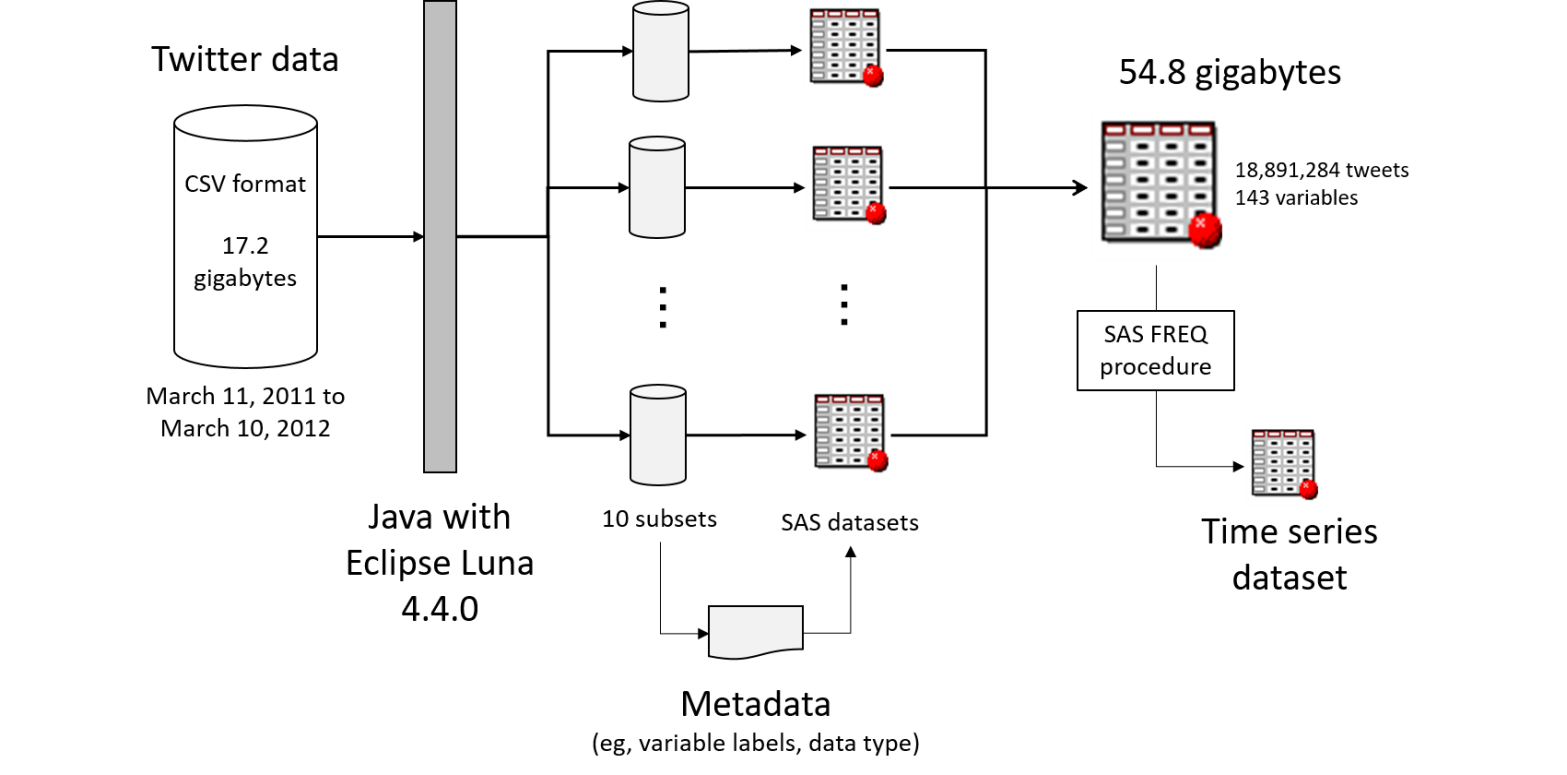
Flowchart showing data construction.

Figure 1 depicts a flowchart for the construction of the dataset. We obtained the Twitter data in CSV format and divided the information into 10 subsets using an original Java program, because it was easy to detect errors when converted into the SAS datasets. To handle the Twitter data using the SAS, we formatted the variable names as alphanumeric. We used the frequency (FREQ) procedure (an SAS analysis program) for count and categorical data, and obtained time series data for the tweet count per day.

We intended to quantify public concern regarding radiation exposure from the Fukushima Dai-ichi Nuclear Power Station accident. Although we treated the tweet count as a measure of public concern, there appeared to be a gap between the concerns of Twitter users and the tweet count. Consequently, we showed the correlation by plotting air dose rate data against tweet count as evidence of surrogacy. Data for the air dose rate around the Fukushima Dai-ichi Nuclear Power Station were available to the public on the Tokyo Electric Power Company’s webpage [28], and as of June 2015 these data had been updated only in Japanese. The Tokyo Electric Power Company established eight monitoring posts (MPs) to estimate the air dose rate within the area of the power station.

This study was approved by the institutional review board of Hokkaido University, Health Science School. Informed consent was waived because all records were anonymized and we surveyed data retrospectively.

### Selection of Keywords Related to Concern About Radiation Exposure

**Figure 2:**
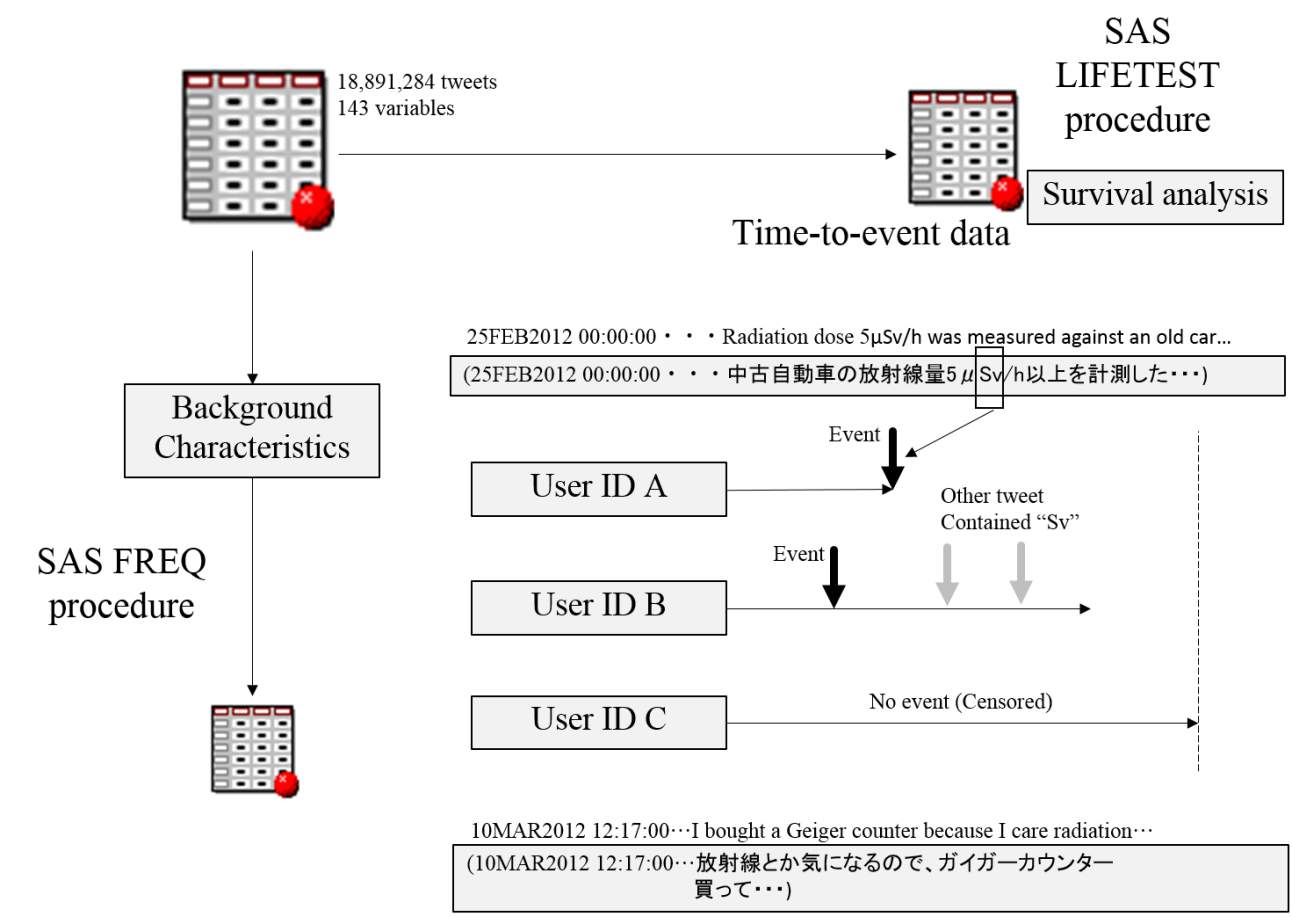
Flowchart for the identification of the primary endpoint.

Keyword selection played an essential role in this research. It was important to select keywords that were sensitive in detecting the concern about radiation risk. Figure 2 shows event detection using the dictionary. When the mass media broadcast information related to radiation exposure, the words *radiation* or *radioactive* were used frequently, along with the radiation units *Sievert* (*Sv*), *gray* (*Gy*), and *Becquerel* (*Bq*). We selected *radiation*, *radioactive*, *Sv*, *Bq*, and *Gy* as keywords to estimate the attenuation period. The keywords *radiation* and *radioactive* were in Japanese and the radiation units were used in their normal format. Natural language processing techniques are frequently used for text analyses in radiology, or to identify patient smoking status; however, it was difficult to merge the two different words into a single concept in the Twitter datasets because the free text part of Twitter data was restricted to 140 characters, leaving little contextual information available to use in this case [29,30]. Thus, we maintained the reproducibility based on the keyword selection using multiple concept names. Using the keywords, we matched the concept names with those terms in the free text portion of the Twitter datasets. The mean proportions of tweets that contained the keywords *radiation* or *radioactive* were plotted on an hourly basis.

When a Twitter user posted a tweet containing one of the keywords for the first time, we regarded this as an *event,* and analyzed it using the SAS LIFETEST procedure. Background characteristics such as mean count per day were plotted as a time series plot using the SAS FREQ procedure. This analysis would normally show a great deal of censored data, so we suppressed the censored plot on the Kaplan-Meier curve.

### Estimating Public Concern Using Survival Analysis

The survival analysis technique is useful for handling and evaluating time series data. In the field of biostatistics, an event in this analysis indicates the death of a subject, and survival time refers to a period with no event (and is thus interpreted as, for example, disease-free survival or progression-free survival). Many textbooks related to survival analyses have been published [31,32], but the use of this methodology in the present study was quite different from its use in biostatistics.

When we conducted the survival analysis, we could not determine the starting time point for the observation because the individual who tweeted the event could not be followed by the authors in social media; this issue highlights one important difference between big data analysis and other types of research, such as clinical trials. We assumed that observation started on March 11, 2011 in a pseudo manner. Although some ambiguity in Twitter data is inevitable, the volume of data can reduce this effect [21]. We assumed the onset of a tweet and defined an event as when the k^th^ keyword occurred in the free text part of a tweet for the first time from a given user ID. Thus, the survival function was computed as follows:

Here, i indicates the i^th^ day after March 11, 2011. Date and time were saved as a continuous variable in SAS. We estimated the duration of concern in seconds. The duration of concern could be attenuated with time; we estimated the mean attenuation period with S(t_ik_).

## Results

### Twitter Data Characteristics

**Figure 3:**
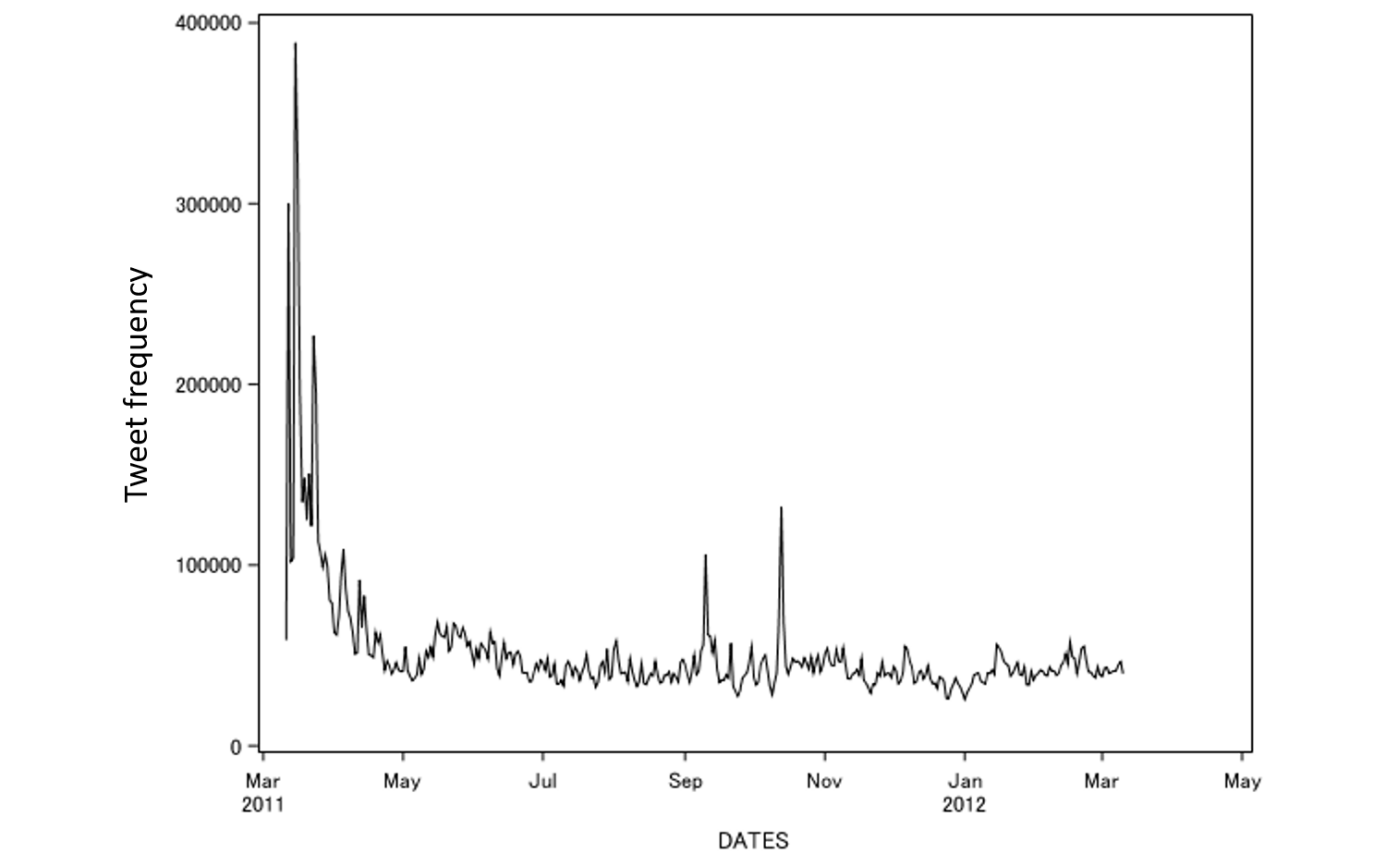
Time series plot of the tweet count over the period from March 11, 2011 to March 10, 2012. The frequency of tweeting peaked at 388,984 per day.

Table 1 shows the characteristics of tweets and Twitter IDs from March 11, 2011 to March 10, 2012. The median tweet count was 43,345 tweets/day, ranging from 25,769 to 388,984 tweets/day. The histogram was skewed right with a skewness of 5.85. The data included 1,168,543 user IDs (16.2 tweets/user ID). Available usernames numbered 879,210, but there were 125,363 usernames that were missing data. We calculated the mean number of IDs per day as 23,626.23, and 2.19 tweets per ID were posted per day. The tweet count per day showed a variation just after March 11, 2011, with spikes in September and October of 2011 in the time series plot (Figure 3). From a total of 18,891,284 tweets, 9,673,756 (51.21%) were retweets of original messages.

**Table 1:** Characteristics of tweets and Twitter IDs on Twitter from March 11, 2011 to March 10, 2012.

	Tweets	Twitter IDs
Total	18,891,284	1,168,543
Mean/day, n (95% CI)	51,616 (48,044-55,187)	23,626 (21,695-25,557)
Standard deviation	34,747	18,786
Skewness	5.85	5.04
Kurtosis	43.15	32.07
Minimum/day, n	25,769	9421
25^th^ percentile/day, n	38,766	15,621
Median/day, n	43,345	18,994
75^th^ percentile/day, n	51,703	23,448
Maximum/day, n	388,984	187,291

The air dose rate showed some variation depending on the wind direction or climatic condition. The MPs output the data for the air dose rate at 10 minute intervals. We downloaded the publicly available data and selected the eight MPs to show air dose rate. Figure 3 shows the mean hourly frequency of tweets plotted for each day of the week. The frequency of tweeting decreased from 4 o’clock to 5 o’clock a.m., and then peaked at 12 o’clock noon. There was a further increase at 8 o’clock p.m. We observed that the overall pattern each day was almost constant, regardless of the day of the week.

**Figure 4:**
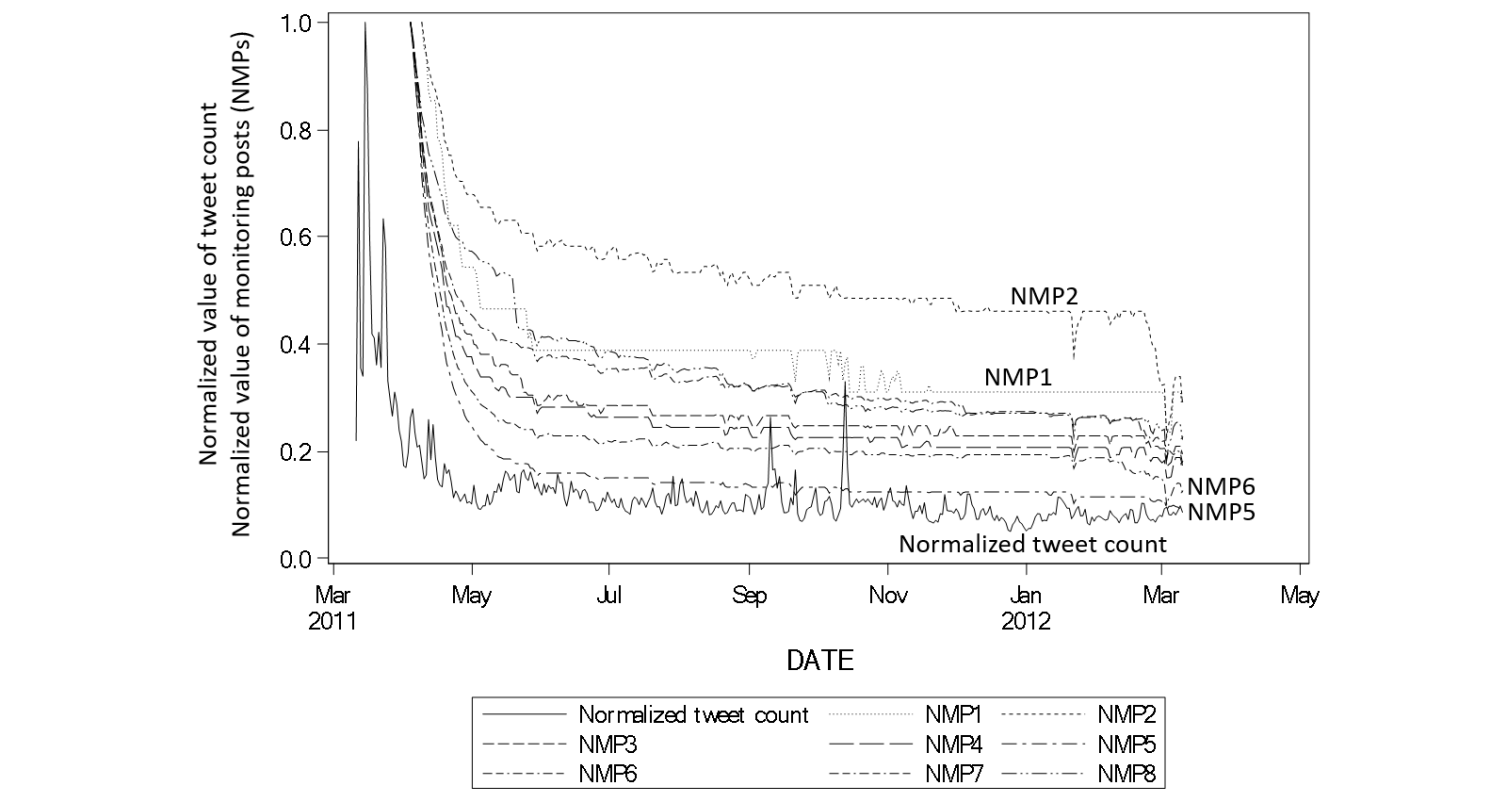
Time series plot of the normalized tweet count from March 11, 2011 to March 10, 2012.

Figure 4 and Table 2 show the similarity in the daily tweet count and the mean MP air dose rate. Figure 4 displays a time series plot of the normalized tweet count over the entire period from March 11, 2011 to March 10, 2012, and the air dose radiation rate measured within the Fukushima Dai-ichi Nuclear Power Station. We observed a peak shortly after March 11, 2011 and relatively small peaks in September and October of 2011; there was also some daily variation. The air dose values observed at an MP were averaged per day, based on the data obtained at 10 minute intervals. Similarity was observed between the air dose rate and the tweet count.

**Figure 5:**
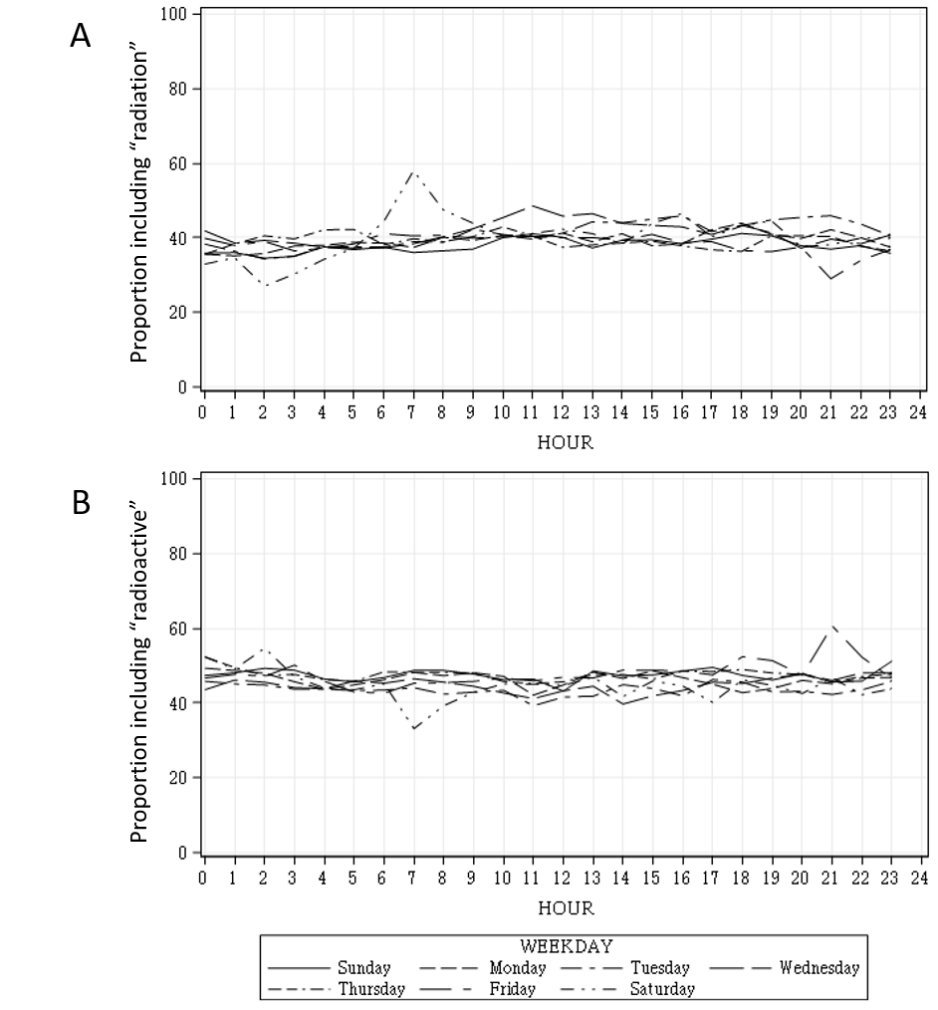
Plot of the keywords radiation and radioactive, including proportion averaged by month for each day of the week.

Figure 5 shows the plot of the keyword, including proportion averaged by month for each day of the week. The proportion plot including *radiation* showed some positive and negative peaks at approximately 7 o’clock a.m. and 9 o’clock p.m. (Figure 5 A); the plot of *radioactive* appeared to be the opposite of *radiation* (Figure 5 B).

**Table 2:** Sum of squared differences between the normalized MPs and normalized tweet counts. The normalized air dose rate of MP-5 and MP-6 showed a good fit to the normalized tweet count plot.

Compared with tweet count	
Monitoring post	Sum of squared difference
MP-1	28.72
MP-2	62.51
MP-3	14.35
MP-4	11.59
MP-5	4.66
MP-6	8.24
MP-7	20.93
MP-8	25.40

### Survival Analysis of Twitter Events: Estimating the Attenuation Period for the Keywords

**Figure 6:**
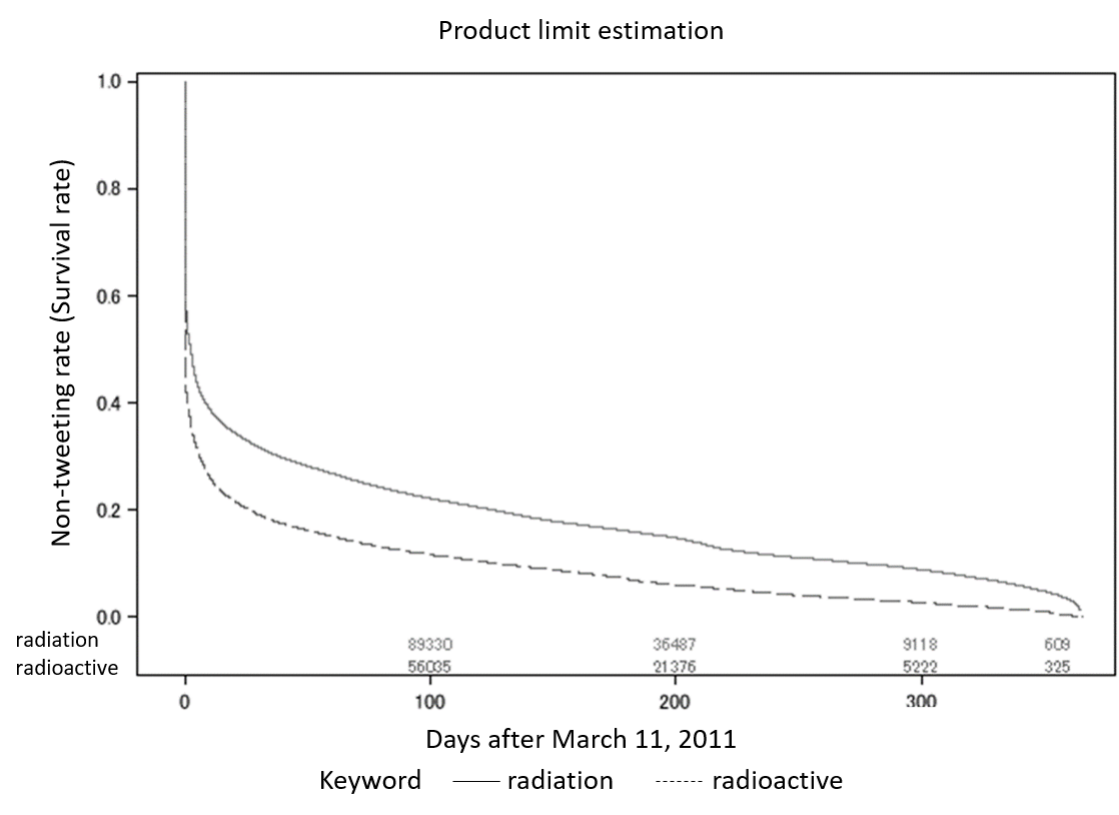
Kaplan-Meier curves of the tweet rates for tweets including the keywords radiation or radioactive.

We conducted survival analyses for tweets that contained the keywords. Figure 6 shows the Kaplan-Meier curve of the first tweet rate for tweets that included the keywords *radiation* or *radioactive*. The curve for *radioactive* dropped below that of *radiation*. People continued to tweet the word *radioactive* a year after the first tweet, and of the 1,168,542 user IDs, 880,108 (75.32%) tweeted *radioactive* (Figure 6). The mean attenuation periods were 63.4 days (standard error [SE] 0.152) and 31.9 days (SE 0.096) for *radiation* and *radioactive*, respectively (Table 3). The mean attenuation period for these was much shorter than those for the radiation unit keywords. The number of events for *Sv* was twice the number for *Bq* and five times that of *Gy* (Table 3). We treated the remaining data as censored.

**Table 3:** Number of events and estimated attenuation period derived from the Twitter data.

Keyword (Japanese)	Twitter ID count	Event	Censor	% Censored	Mean, days	95% Lower Control Limit	95% Upper Control Limit
radioactive (放射能)	1,168,542	880,108	288,434	24.68	31.9	31.7	32.1
radiation (放射線)	1,168,542	710,924	457,618	39.16	63.4	63.1	63.7
Sv	1,168,542	107,522	1,061,020	90.8	300.6	300.2	301.0
Bq	1,168,542	53,034	1,115,508	95.46	330.3	330.0	330.6
Gy	1,168,542	17,111	1,151,431	98.54	354.6	354.4	354.8

**Figure 7:**
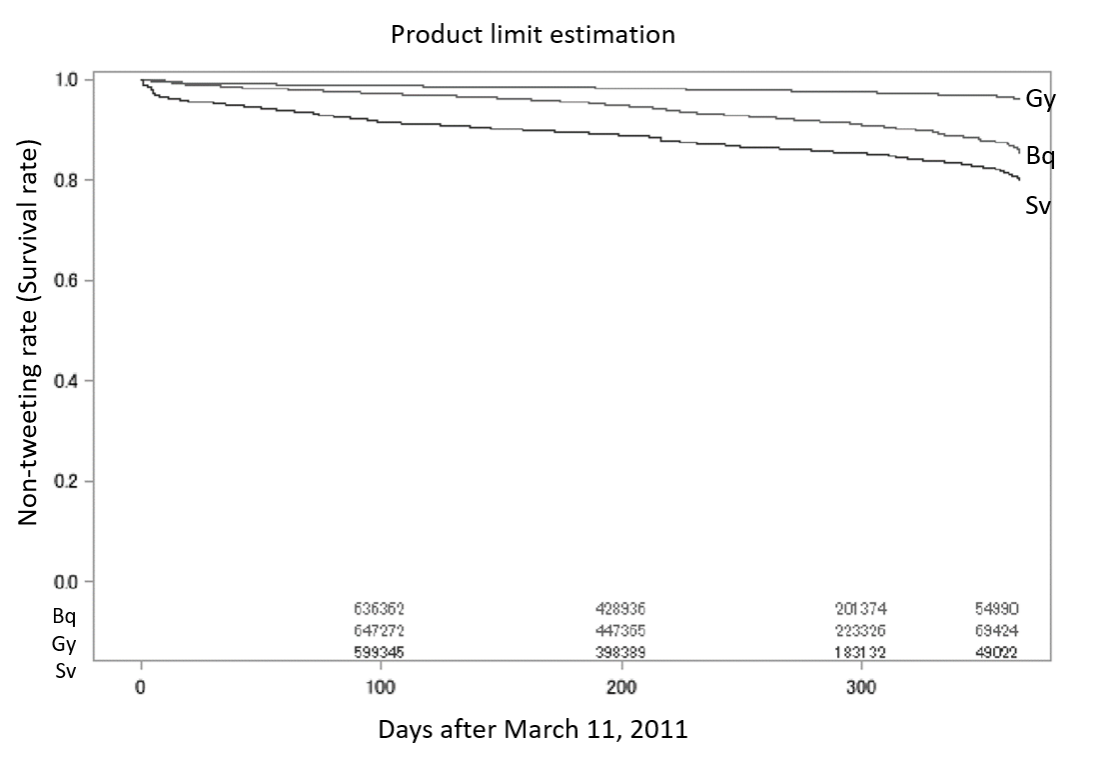
Kaplan-Meier curves of the tweet rates of tweets including the keywords Bq, Gy, or Sv.

Figure 7 shows the Kaplan-Meier curve of the first tweet rates for tweets that contained the keywords *Sv*, *Gy,* and *Bq*. Users continued to tweet messages that included *Sv* up to one year after the first tweet. Of the 1,168,542 user IDs, 107,522 (9.20%) tweeted *Sv*. We were not able to obtain the median for the first tweet rate between March 11, 2011 and March 10, 2012 because the event rate decreased to approximately 0.8 within one year.

We observed a steep decrease in nontweeting rate during the first 7 days after March 11, 2011 (Figure 6). This finding indicated the occurrence of automatic tweeting from bots. Tweets by bots can be identified from their contents; however, no specific data handling was carried out for these user IDs because natural language processing is arduous in this context, and we decided that bot identification was beyond the of scope in this study. There was a gradual decrease in nontweeting rate for tweets that referenced radiation units, and no sudden changes were identified during the observation period in (Figure 7). Table 3 shows the counts for the events and censored data.

## Discussion

### Tweet and Twitter ID Trends Became Stable in the Count Plots

The maximum number of Twitter IDs tweeting a keyword in a single day was approximately 187,000. At its minimum, the number of Twitter IDs was 9,421 per day (approximately 5% of the maximum), while the median was 18,994 per day (approximately 10% of the maximum). However, the 75^th^ percentile (23,448 IDs/day) was not greatly different from the median (Table 1). We observed that the maximum number of IDs per day was an outlier.

Although the count of Twitter IDs did not directly indicate the number of users, only 1.00% of the Twitter users appeared to be bots (Table 4). The effect of bots could be estimated, and we decided that no specific data handling would be given to tweets from suspected bots. We could not establish the Twitter share in social media among the population; however, it has been estimated that 79.1% of the population of Japan were Internet users in 2011 [33]. We can therefore conclude that the survey from Twitter showed a trend pertaining to the entire population.

**Table 4:** Percentiles of tweet counts per Twitter ID (N=1,168,542).

Level	Percentile	Number (n)
Maximum	100%	71,793
	99%	243
	95%	42
	90%	18
3^rd^ quartile	75%	6
Median	50%	2
1^st^ quartile	25%	1
	10%	1
	5%	1
	1%	1
Minimum	0%	1

### Time Series Plot and Combination with Other Epidemiological Data

The time series plot of tweets (Figure 3) showed a surge just after March 11, 2011, and then a decrease until May 2011, after which time the number of tweets appeared to be stable. We observed some daily variation, and two peaks from September to November of 2011. We assumed that the number of tweets tended to follow the decrease in the radiation dose (Figure 4); however, it was difficult to estimate the effects of mass media. We assumed that public concern was burned out immediately, despite that the fact that the risk of radioactive exposure persisted. The Tweet counts may have decreased because Twitter users did not live near the Fukushima Dai-ichi site, but further study would be required to determine the location of the users.

When we examined the event related to the surge of tweets on September 10, 2011 (Figure 3), we discovered that the Japanese Minister of Economy, Trade, and Industry made a negative comment about the Fukushima Dai-ichi Nuclear Power Station accident in an interview, and the Japanese mass media broadcast this statement. Contents of Twitter data at that time indicated that many tweets mentioned or retweeted this news story. On October 13, 2011, we found that the tweet count increased because a sealed radioactive source was found under a house in Tokyo, and the mass media broadcast this news. However, this incident was not directly related to the Fukushima Dai-ichi Nuclear Power Station accident, although Twitter users still interpreted it as news about radiation exposure.

The time series plot in Figure 3 indicated that the tweeting trend was somewhat ambiguous as it related to how many users were actually concerned about radiation exposure. From another point of view, we observed that a simple plot of the tweet count indicated that public concern was high at first, and then decreased and became stable.

### Estimating the Attenuation Period

We observed that the first tweet counts differed in number, with *Sv* being mentioned the most, followed by *Bq* and *Gy*. These counts indicated that Twitter users observed mass media using the radiation unit *Sv,* which is related to the effects of radiation exposure on humans. Approximately 1.00% of Twitter user IDs posted a large number of tweets related to our keywords (more than 200 tweets over the year); we checked the contents of these messages and determined that they had the traits of auto-posted messages, such as daily radiation reports. To estimate the activity from these indexes, the problem of nonhuman user IDs remained. Some researchers have reported using natural language processing techniques based on the contents of tweets; however, it remained difficult to identify nonhuman users [24,25].

There appeared to be another reason for the difference between the mean and median: some IDs posted no message during the observed period. The mean was therefore assumed to be considerably reduced, but a sensitivity analysis would be required to clarify this issue. To predict the changes in a trend over a period of time, cyclic variation reduction methodology is required from another domain, such as time series analyses.

We could not estimate the median attenuation period from the Kaplan-Meier plot. However, we estimated the nonfirst tweeting rate (survival rate) at 365 days after March 11, 2011. A tweet (containing the keyword) that was the first for that user ID was observed, and there was a reduction in the nonterm tweeting rate within the first 7 days. The reduction at this time point was highly dependent on the users that we identified as bots. We did not exclude these in our research because this led to an overestimation bias of the nontweeting rate. Figure 6 and Figure 7 show that the nontweeting rate decreased gradually with time. In reference to the time series plot in Figure 3, only a small number of tweets were shown in the plot, and the trend showed a steep peak within the first 7 days, followed by a stable trend. When we focused on the Kaplan-Meier plots (Figure 6 and Figure 7), we clearly observed that the tweet that mentioned keywords for the first time continued to be posted for a year.

Figure 6 and Figure 7 show that the rate of user IDs that posted a keyword increased one year after the Fukushima Dai-ichi Nuclear Power Station accident. The simple time series plot in Figure 3 showed the peak immediately after the accident and the subsequent decrease in tweet count, demonstrating that public concern became stable. However, Twitter users had fears or concerns about nuclear exposure, as seen in the event probability plot (Figure 6 and Figure 7). When we drew a time series plot of the tweet count, the information related to public concern was hidden. We must consider that not every tweet expressed a negative attitude towards radiation exposure, although public concern could be a denial of radiation exposure. Nuclear power plant activity ceased throughout Japan after the accident at Fukushima, and we believe this fact supports tweet contents that reflected a negative attitude towards nuclear energy. Thus, using the event probability plot by time series was a powerful technique for this purpose.

### Limitations

We used Twitter data to detect public concern about radiation exposure; however, some ambiguity was shown in our study. If we had been following the techniques of surveillance in an epidemiological study, we should have identified each participant in the study and followed them with a time series. We had Twitter user IDs, and these could be shared or checked by an automated program. We assumed that this fact is not accepted in the epidemiological domain, although we would like to emphasize the effectiveness of this method for early stage prediction and the quantification of public concern about events in society.

In addition, we tracked the radiation-related keywords, but we did not consider the context in which they were used. The positivity or negativity of the tweets should be added into the analysis, which would allow for greater precision when analyzing the data.

### Future Work

We selected very few keywords for our analyses, and the estimation of Twitter users’ interest had some limitations. Natural language processing could be used for content analysis as an engineering task. Similarly, direct emotions (eg, concern or fear) could also be challenging in the health care domain. The effect of low dose radiation exposure was not clear in the present study. We would like to quantify these emotions as an effect, in addition to the biological effects on human health. Using survival analysis is still problematic because the definition of onset was not clear, and this may be difficult to accept as an observational study in the epidemiological field. Further study is required to adapt the current methodology.

### Conclusions

Although the simple plot of the tweet count did not show clear results, we estimated the mean attenuation period to be approximately one month for the keyword *radioactive,* and found that this keyword was still being used at the end of the investigation period. Further research is required to quantify the effects of other phrases in social media data. The results of this exploratory study should advance progress in influencing and quantifying the communication of risk.

## Abbreviations

ASA: American Statistical Association
Bq: Becquerel
CSV: comma-separated values
FREQ: frequency
Gy: gray
ID: identification
MP: monitoring post
SAS: Statistical Analysis System
SE: standard error
Sv: Sievert
